# Adaptive noise

**DOI:** 10.1098/rspb.2013.1104

**Published:** 2013-09-22

**Authors:** Mark Viney, Sarah E. Reece

**Affiliations:** 1 School of Biological Sciences, University of Bristol, Woodland Road, Bristol BS8 1UG, UK; 2 Centre for Immunity, and the Infection and Evolution, Institutes of Evolution, Immunology and Infection Research, School of Biological Sciences, University of Edinburgh, Edinburgh EH9 3JT, UK

**Keywords:** bet hedging, evolution, variation

## Abstract

In biology, noise implies error and disorder and is therefore something which organisms may seek to minimize and mitigate against. We argue that such noise can be adaptive. Recent studies have shown that gene expression can be noisy, noise can be genetically controlled, genes and gene networks vary in how noisy they are and noise generates phenotypic differences among genetically identical cells. Such phenotypic differences can have fitness benefits, suggesting that evolution can shape noise and that noise may be adaptive. For example, gene networks can generate bistable states resulting in phenotypic diversity and switching among individual cells of a genotype, which may be a bet hedging strategy. Here, we review the sources of noise in gene expression, the extent to which noise in biological systems may be adaptive and suggest that applying evolutionary rigour to the study of noise is necessary to fully understand organismal phenotypes.

## 1. Difference among the same

It is axiomatic that genes control phenotypic traits, so that genetically identical individuals in the same environment will be phenotypically identical. Clearly, this is continuously falsified; genetically identical human twins are not quite phenotypically identical; genetically identical fruit flies often differ in trait values. The observation of differences among genetically identical individuals in a common environment is long standing, for example, apparently non-genetic variation among populations of bacterial cells in homogeneous environments [1]. Such differences among genetically identical individuals are usually thought to be owing to some small, chance differences in conditions and errors that occur as individuals develop. Intuition suggests that this variation will move phenotypes away from the optimum, and so will be selected against. Indeed, canalization and homeostasis are phenomena that generate similarity and maintain stability in the face of environmental variation.

Another view of phenotypic differences among genetically identical individuals is that it is an inevitable feature of how phenotypes are produced and controlled. Indeed, theoretical studies have shown that costs limit the production of highly precise, non-noisy biological systems and that such costs may constrain the evolution of such systems [2]. For example, among-individual variability can be generated simply by the distribution of low numbers of molecules among individual cells. Low concentrations of molecules will be subject to Poisson-type (i.e. near random) distributions, so that cells will differ in the number of molecules that they contain [3]. When a molecule's concentration has phenotypic consequences then variation in the number of molecules each cell receives will result in phenotypic heterogeneity among genetically identical cells, in the absence of environmental variation [1].

Recent progress in understanding the sources of phenotypic heterogeneity has focused on noise in gene expression, specifically measuring gene expression within and between genetically identical cells [4]. Phenotypes are the product of genes and gene networks which are themselves noisy, and it is these phenotypes that natural selection selects for, or against. The phenotypic heterogeneity caused by noise in gene expression can affect reproduction, survival and hence the evolutionary fitness of a genotype. Furthermore, observations suggest that noise, and the variation that it can generate among individuals of one genotype, is heritable. Therefore, all of the ingredients required for noise to be shaped by evolution exist, that is a heritable mechanism that generates phenotypic variation in fitness-related traits.

With this perspective noise may, under some circumstances, be beneficial, and here our aim is to explore the potential of noise to be adaptive. In so doing a rigorous evolutionary framework needs to be applied to noise and its potential adaptive value.

## 2. Noise in gene expression and gene networks

**Figure 1.**
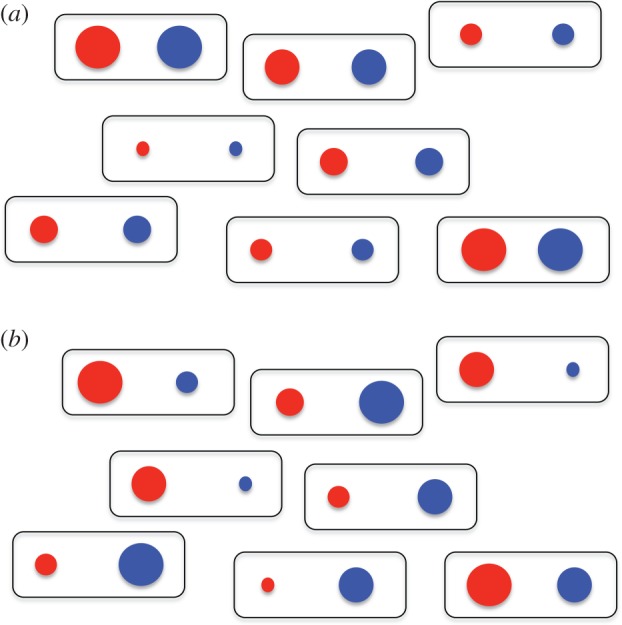
The expression of two genes (one red and one blue), among cells of one genotype, where circle size is a measure of expression. (*a*) Extrinsic noise is where there is similarity of expression of these genes within each cell but differences among the cells. (*b*) Intrinsic noise is where there is difference in expression of these genes within each cell.

For any gene, the quantity of protein it produces can vary among cells: this is called noise, measured as the coefficient of variation of the quantity of protein (box 1). Noise can be extrinsic or intrinsic. Extrinsic noise is that which is common to genes of any one cell, due to differences between cells, such as energy state, or concentration of regulatory molecules, etc*.* [7]. Intrinsic noise is that which is specific to any one gene, for example because of transcriptional and translation effects. By examining cells containing two reporter genes, where the reporter genes' products can be distinguished [4,7,8], these types of noise are manifest; extrinsic noise is the similarity of expression of these genes within a cell, but there may be differences among cells; intrinsic noise is differences in expression between these two genes within a cell (figure 1 and box 1). For example, in *Escherichia coli* both extrinsic and intrinsic noise make a substantial contribution to among-cell heterogeneity; intrinsic noise is greater at low transcription rates (consistent with earlier ideas about low molecular concentrations [9]), and a cell's genetic background also affects the amplitude of noise [4]. Similar findings have also been made in eukaryotes [8,10]; indeed, it now appears that noise is common among diverse systems—it is likely to be a universal feature of life [9].

Box 1. **Glossary.**

*Noise*. Random or irregular fluctuations in a signal of interest. For expression of genes, noise is therefore variation in that expression. For a population of identical cells in a homogenous environment, differences among cells in their expression of a gene is the noise of that gene's expression [4]. Noise has both a frequency and an amplitude.
*Extrinsic and intrinsic noise*. This was first defined by considering two distinguishable genes within a cell, among a population of genetically identical cells in a homogenous environment [4]. Extrinsic noise is the noise in gene expression that is common to the two genes within any one cell but different between cells. Intrinsic noise is the noise in gene expression that is specific to one of these two genes within a cell (figure 1).
*Gene expression*. Strictly, the generation of mRNA molecules from a gene by the process of transcription. More generally, the generation of protein products from genes.
*Gene networks*. A collection of genes that interact typically by the product of one gene affecting other genes in the network. These networks, or circuits, can therefore have features seen in other networks, such as feedback loops, etc*.*
*Bistabilty*. When there are two stable states. Applied to gene expression states, then these alternative states exist across otherwise identical conditions [5].
*Fitness*. The evolutionary success of a genotype. Natural selection selects individuals with the highest fitness. In experimental evolution (below), the fitness that is selected is defined by the experimenter. Fitness can be conceived of as an absolute quantity or as relative quantity.
*Arithmetic and geometric mean fitness*. Natural selection selects on fitness across multiple generations. Across-generation fitness is therefore a multiplicative process, which can be captured by calculating the geometric mean of fitness of an individual. Arithmetic mean fitness is another way in which across-generation fitness could be calculated, but it does not capture the multiplicative nature of multi-generational fitness. Consider two individuals’ reproductive output over four breeding seasons. A = 10, 30, 50, 30; B = 15, 29, 42, 29. By arithmetic mean fitness A > B (30 versus 28.75, respectively), but by geometric mean fitness B > A (26.98 versus 25.90, respectively).
*Phenotypic plasticity*. The process by which one genotype can generate different phenotypes in response to the environment [6]. Plasticity of gene expression is therefore when the environment causes a change in the expression of a gene.
*Bet hedging*. Strategies that maximize geometric mean fitness at the expense of arithmetic mean fitness. While many aspects of life histories are described as bet hedging strategies, rigorous proof of this (which requires multi-generational measurement of fitness) is often wanting.
*Experimental evolution*. Studying evolution in controlled conditions, particularly by artificial selection experiments. This is commonly used with either microorganisms or other species with short generation times.

The details of transcription and translation contribute to intrinsic and extrinsic noise. These processes can be modelled such that a gene changes between ‘on’ and ‘off’ states, with certain time intervals between the initiation of different transcription events, and from which probability distributions of subsequent events are constructed [11] (for a detailed review see [3]). Details of the control of these processes, for example how feedback loops act (e.g. on transcription or on translation, and whether this depends on the concentration of mRNA or protein), have different effects on the noise of gene expression [12]. However, an important, general conclusion that comes from these models is that transcriptional and translational bursting (i.e. many transcripts result from one transcriptional ‘on’ state, and many protein molecules are produced from one mRNA molecule, respectively) increase the noise in gene products present and, further, that the balance of production of proteins from mRNA molecules, and the decay of the mRNA molecules also contributes to noise in gene expression [9,11,13].

Multi-locus effects contribute to noisy gene expression too. This is seen as differences in the noise in gene expression among different genotypes and as the noise-increasing effects of *cis*- and *trans*-acting mutations [1,4,14,15]. Such studies have been experimentally extended genome-wide, for example by analysing all of *E. coli* promoters' functions in the production of a reporter protein [16]. Several general patterns emerge: (i) that there is more noise at low levels of expression (again in line with earlier predictions [1,3]), (ii) that, by comparing expression among promoters of different classes of *E. coli* genes, so-called essential genes have less noisy gene expression (after correction for the average level of expression) compared with other genes, but (iii) that promoters of genes in different functional classes (e.g. DNA configuration, amino acid biosynthesis, energy metabolism, etc.) differ in their noisiness [16]. In *Saccharomyces cerevisiae*, gene promoters can affect noise [17]; recent studies have found that genes with high extrinsic noise have common promoter motifs, such that members of pathways share extrinsic noise expression [7]. This could explain why genes of different functional classes have shared extrinsic noise of gene expression [7].

Genes act within gene networks, and this therefore raises questions of whether noise is propagated through a network, and what effect noise has on the output of a network [2,7,8,10,18–21] (box 1). Noise may play a functional role in gene networks. For example, some gene network structures (e.g. negative autoregulation) enhance noise frequency, which (perhaps counterintuitively) may enhance the ease with which noise can be filtered out [19]. In a *Bacillus subtilis* gene network, noise plays a key role in generating pulsed circuit outputs (which have further downstream transcriptional effects) from sustained, non-pulsed circuit inputs [22]. The structure of a gene network can also affect how noisy a circuit is. Comparing a native gene circuit and a modified circuit (by inversion of positive and negative regulation loops) of *B. subtilis* revealed that both had the same basic function, but that the modified circuit was less noisy (and differed in other functional parameters too) [23]. The noise difference in the circuits was owing to differences in the concentration of a central molecule acting within the circuit; the noisy native circuit had a lower concentration [23]. Thus, gene circuit structure affected the quantity of molecules present, from which noise arose *via* stochastic events on those comparatively few molecules.

## 3. Can noise evolve?

Considering all of the findings from different study systems together, it is clear that low levels of gene expression are inherently noisy, but beyond this, noise can differ among genes. Features of individual genes (particularly those controlling expression) as well as the gene network within which a gene functions both contribute to the noise of a gene's expression [9]. Moreover, noise in any one gene's expression can be functionally eliminated by a gene network or be amplified and fed-on to other networks. If the output of a genetic network is an organismal behaviour, trait or process that affects fitness, then the underlying noise in gene expression and in gene networks is subject to selection (either to reduce or enhance noise). It is also important to be aware that noise may also be selectively neutral (i.e. neither selected for or against), in which case it would evolve by drift. Clearly, gene expression itself evolves [24,25] and so mechanisms leading to differences in the noise of gene expression can also be the result of natural selection [10]. For this to occur, noise in gene expression must be heritable. There has only been one explicit study of the heritability of noise, which measured the noise of expression of a construct introduced into segregants from a cross between parental *S. cerevisiae* strains [14]. Beyond this, there are three types of evidence which implicitly support the idea that noise is heritable; (i) individual genes differ in their noise of gene expression and that the noise of a gene's expression can be changed by genetic manipulation or alteration of gene networks; (ii) bistable states, which we argue below are underpinned by noise, are inherited in bacterial lineages and (iii) variance in phenotypes can be genetically controlled and selected on [26,27].

If we consider noise in gene expression as a trait, then gene-specific effects as well as gene network structure can alter this trait. The observation of a relationship between noise in gene expression and certain classes of gene (classified by the likely function of their products) may therefore reflect the evolutionary history of natural selection acting on noise. In this view, noise in gene expression is not necessarily a stochastic process which evolution cannot alter, nor is noise necessarily deleterious and something which natural selection seeks to reduce, minimize or eliminate [21]. However, to understand how natural selection may shape such noise of gene expression requires more effort to quantify its fitness effects.

## 4. Can noise affect fitness?

Noise in gene expression within gene networks contributes to organismal traits and phenotypes, which themselves contribute to fitness (box 1). It is therefore self evident that noise in gene expression can affect fitness, but its precise role or the magnitude of its effects is unclear. Very few studies have investigated the fitness effects of the noise in gene expression directly, and those that have, have so far mostly used single-celled species. For example, when genotypes of *S. cerevisiae* with high or low noise in gene expression were compared, each was advantageous but under different environmental conditions, specifically, increasing concentrations of antibiotic; the strain with more noisy gene expression performed better at high concentrations [20]. A corollary of this observation is that selection in these different environments could result in *S. cerevisiae* genotypes with either high or low noise in gene expression. In a second *S. cerevisiae* study, noise within a gene circuit caused a bimodal distribution of gene expression among cells, that ultimately resulted in inter cell heterogeneity in sensitivity to a mating pheromone within a pheromone gradient [28]. This heterogeneity generated diversity in commitment to mating, which is likely to have some effect on fitness [28]. Here, noise is a force that generates phenotypic diversity within a genotype and is subject to natural selection owing to its fitness consequences. Other studies in *S. cerevisiae* have concluded that for genes where a change in their expression affects fitness, those genes have evolved to be comparatively less noisy in their expression, whereas other genes can be noisy in their expression without having fitness costs [29].

Together, while these are laboratory studies (so the relevance to the natural world remains to be determined), they suggest there are clear context-dependent benefits of different noisiness of gene expression. However, it is also clear that there needs to be wider investigation of the phenotypic consequences of differences in the noise in gene expression. This requires testing genotypes' components of fitness in different environments and selecting genotypes in these different environments to determine how (if at all) their noise in gene expression has altered. Furthermore, the studies outlined above have considered the direct fitness effects of noise, but noise can also affect fitness indirectly, which we consider below.

## 5. Noise can be plastic

So far, we have considered the noise in gene expression, including its fitness consequences, that occurs under static conditions. However, both gene expression and phenotypes can respond to changes in the environment; they are said to be phenotypically plastic [6]. Phenotypic plasticity is common and is a way in which an organism can maintain fitness as its environment changes (box 1). The relationship between how noisy the expression of a gene is, and the plasticity (i.e. the sensitivity to a change in the environment) of expression of a gene or its product has begun to be investigated. For example, *E. coli* promoters that were able to drive plastic gene expression were found to be no more noisy than promoters driving non-plastic expression [16]. In *S. cerevisiae*, though, environment-responsive genes appear to be more noisy compared with other gene classes [30]. Furthermore, this varied among the types of promoters that genes have, such that for some promoter types this relationship is strong, for others very weak [17]. A high proportion of genes have context-dependent expression, with expression controlled by the organism's external and internal environment. The broader role of noise in the control of this variable gene expression is largely unstudied, and clearly further work needs to be done in this area.

Phenotypically plastic traits depend on their environment. In the language of quantitative genetics, they are environmental (E) rather than genetic (G) effects. For classical phenotypically plastic traits, this is seen, for example, by placing genetically identical individuals in different environments and observing the resulting phenotypic differences. However, noise cannot be attributed simply to E or G effects, because noise can generate phenotypic differences among genetically identical individuals within the same environment; that is, both G and E are constant. The resolution of this apparent conundrum is to consider each individual cell as having a unique microenvironment. Therefore, for genetically identical cells in an apparently homogeneous environment, the microenvironment of each individual may actually be unique (an equivalent situation in other settings refers to individual (I) effects [31]). Further, this microenvironment could be the cell's external environment *per se* and/or it could be the cell's internal environment, or state. Therefore, for a phenotypically plastic trait, the environment needs to be considered at different scales. It can be common to all cells, to a subset of them or unique to each cell. Moreover, at this smallest scale, the environmental effect can range from the overall environment inside a cell, through to individual genes (and their products) within a network. Therefore, at this scale, phenotypic plasticity effects may be equivalent to extrinsic (common to genes of a cell) and intrinsic (unique to a gene in a cell) noise. That noise can have a role in plastic responses therefore expands the extent to which noise can affect traits, and thus brings such noise to a more central position in considering the control of fitness-related phenotypes.

## 6. Noise has a role in decision-making and bistability

Cells have to make decisions, and variation in a cell's external or internal environment can result in noise in the information that cells use to make such decisions. The problem of noisy input information is analogous to a within-cell gene network which makes an output decision when inputs to, or components of, the network are noisy. Decision-making based on input signals applies both to unicellular organisms sensing their external environment or internal state, as well as to individual cells within multicellular organisms responding to intercellular signals. In all cases of cellular decision-making, simple threshold systems could exist, that is if the intracellular concentration of a molecule exceeds a threshold, it is assumed that the extracellular concentration is of the required concentration to execute a cellular action. However, such thresholds perform poorly if the molecular concentrations (both internal and external) fluctuate [32]. Theoretical studies have examined the accuracy with which a cell can use information about its internal state to correctly predict the external environment and found that more highly cooperative networks (and those with negative inference systems) performed best [33].

Gene networks can exist in bi- or multi-stable alternative states which can lead to bi- or multi-stable phenotypic states [5] (box 1). Bistability phenomena can therefore be used to make cellular decisions, creating phenotypic differences among otherwise genetically identical individuals [34]. If a network is ultrasensitive (because of high cooperativity) to an input and if this is coupled with positive feedback, then this can lead to bistability [35]. These ultrasensitive thresholds (on–off; off–on) are hysteretic, that is that their response to an intermediate input depends on the cell's history. This can also have the consequence that reversion of a cell's state is prevented even if the input state changes, which can be thought of as cellular memory [36]. Detailed investigation of one *S. cerevisiae* network has shown that its memory state can be tuned, for example by changing the network structure as well as the values of network inputs [36]. Specifically, this network had three feedback loops, and systematic removal of these produced these different network behaviours.

With respect to bistable states, noise is a positive force, because it can play a role in cellular decision-making. This is true both for microbes and during development in multi-cellular organisms. However, in other settings, the effects of noise have to be avoided. Organisms therefore have a spatial and temporal context of the utility of noise. For example, developmental processes require that differences are generated on which to build embryological pattern [34,37,38]. In these cases, noise within the system is important because it allows cells to make correct decisions. The contrast is that once a decision has been made by a cell, in this case, a cell of an embryo committing to a developmental fate, then that fate has to be executed with high fidelity, such that noise in this process would be damaging [21,37]. Bi-stable systems are one way in which a decision without reversion can occur, because in such systems, it is hard to move from one of the stable positions; put another way, the system has a memory of the decision that was made [37]. Alternatively, making a decision and executing the decision could be controlled by different gene networks and the latter could be a network that is non-noisy and/or unsusceptible to gene expression noise. Analogous arguments about decision-making and fidelity of decision execution also apply to phenotypically plastic responses. Here, a cell or an organism has to make a developmental response or decision based on noisy environmental inputs, but once a decision is made (e.g. morph A versus morph B), it has to be executed accurately and without reversion [21]. Thus, noise could be selected for in a wide range of processes that facilitate cell decision-making and selected against during the execution of such decisions.

Genetics and developmental biology have progressed by studying the phenotypic effect of mutations. One common observation is that a mutant phenotype is incompletely penetrant; that is, some of the individual mutants have the mutant phenotype, whereas others have the wild-type phenotype. This is another example of where genetically identical individuals (i.e. they all have the same mutation) have different phenotypes (i.e. some are mutant, some wild-type). There is evidence that noise plays a role in this phenomenon. In the model nematode *Caenorhabditis elegans*, a mutation that affects the morphological development of intestinal cells is incompletely penetrant owing to noise [39]. Specifically, this phenotype is controlled by a small transcriptional network, but the mutation increases noise in one part of the network that uses a molecular threshold causing both mutant and non-mutant phenotypes, i.e. incomplete penetrance [39,40]. Presumably, therefore, in wild-type genotypes, the degree of noise has been reduced by selection for a signal sufficiently far from the threshold within the transcriptional network. Other studies have shown that overexpression of a transcription factor can increase penetrance [41]; similar effects are seen between ancestrally duplicated genes coding for transcription factors in the *C. elegans* genome [42]. Here, the effect of the transcription factor reduces the noise in gene expression, thereby reducing differences between individuals and increasing penetrance. Again, extending this to the wild-type state, the level of transcription factor-driven noise in gene expression may have evolved to generate the wild-type phenotype.

## 7. Bistability has fitness consequences

There has been investigation of the fitness effects of bi- (or multi-) stable states, which result in a genotype generating diverse phenotypes, in which each phenotype matches one of the environments likely to be encountered [32,35]. Theoretical studies have identified environmental regimes where such behaviour can evolve [43]. We have already considered bi- and multi-stable states of gene networks which can result in bi- or multi-stable phenotypic differences among individuals. It is also clear that bi- or multi-stable phenotypic states can occur without underlying bi- or multi-stable network systems [44,45]. In bacteria, examples of such bistable states and phenotypes are persister cells (e.g. cells that survive in the presence of antibiotic, but not because of mutation-dependent antibiotic resistance), genetic competence (the ability to take-up DNA from the environment), sporulation (formation of a dormant spore phenotype), and swimming and chaining (alternative phenotypes where some cells remain together after cell division, looking like chains, whereas other individual cells are free and swimming) [5]. There are many ways that phenotypic diversity can be generated, but we focus on bi- (or multi-) stable states because these provide clear examples that relate to noise in gene expression.

The generation of apparently random differences among identical individuals can be thought of as a strategy that enhances fitness, compared with a mono-phenotypic strategy [32]. In principle therefore, a genotype could be selected for such a bi- or multi-stable phenotype, and the generation of this phenotype may be achieved by noise in gene expression. There is experimental evidence that differences among identical individuals can be advantageous. Selection of a *Pseudomonas fluorescens* monomorphic genotype through different, alternating environments, produced genotypes with apparently stochastic switching in colony morphology, and this had a large fitness benefit in the alternating environments [46]. Genetically, this switching phenotype appeared to require a series of mutations, which may generate a bistable state, though this was not investigated directly [46]. Importantly though, this work shows that selection can change a genotype from a mono-phenotypic form to a genotype with multiple-phenotypic forms. In *Salmonella* sp., a bistable virulent/avirulent phenotypic mixture exists that checks the evolution of avirulent cheating genotypes [47].

Experimental studies have also investigated how genotypes that produce individuals of two phenotypic states perform in different environments. Comparison of *B. subtilis* genotypes with noisy or non-noisy gene circuits (resulting in different durations of competence) performed similarly in some environments, but the phenotype of the more noisy circuit genotype had a superior phenotype in other environments [23]. Experimental comparison of two *S. cerevisiae* genotypes that, because of noise in gene expression, switch between two alternative phenotypes at different rates found that each genotype had greater fitness among switches in environmental conditions (where each environment favoured only one of the *S. cerevisiae* genotypes) [48]. Recently, it has been shown that *S. cerevisiae* has a continuous phenotypic distribution (specifically, cellular growth rates caused by different among-cell noisiness of gene expression), which appears to provide a continuum of different fitness in a given environment [49]. This is an example of the fitness effects of noise, but where bistability *per se* may not be involved. In all of these cases, noise in gene expression affects the rate of production of phenotypic difference among individuals within a genotype and this phenotypic diversity among individuals (and, presumably, the frequency distribution of that) gives different fitness benefits in different environments. Overall this appears to be clear evidence that noise in gene expression is at least part of the means by which organismal fitness (*via* the phenotypic diversity generated within a genotype) can be controlled.

## 8. Bistability in variable environments

In the examples above, the phenotypic switching occurs automatically, not in response to an environmental signal and so these processes do not fit the traditional definition of phenotypic plasticity. This phenotypic switching is, though, the result of selection in the environments in which that genotype evolved. It therefore appears that, in effect, the frequency with which the genotype encountered the different environments has been encoded in the genome via a fit response to this. Selection is therefore acting on a genotype to produce multiple phenotypic states among individuals of that genotype. Importantly, this is different from true randomness or stochasticity (if such a thing exists) in phenotypic switching as outputs of genetic networks. The examples of phenotypic switching considered above should therefore not automatically be thought of as random switching but may instead be phenotypic switching that is the precise and honed result of evolution. That said, theoretical studies show that strategies of producing random difference are best when environmental information is poor, absent or too costly to process [32].

It is not clear from observation alone how one can differentiate between a random strategy of phenotypic switching and a tightly controlled strategy of phenotypic switching that has evolved specifically to match the rate of encountering different environments when each occurs with equal frequency. Experimentally, a genotype with a random strategy of phenotypic switching may maintain its fitness under a wide range of different randomly encountered environments. By contrast, a non-random strategy of phenotypic switching may have maximal fitness under one series of environments (those in which it was selected) but a loss of fitness in other environmental switching regimes. Because all that can be observed is the frequency of phenotypic switching, experimental evolution approaches could be used to explore how different selection regimes act on phenotypic switching rates. An example would be to test whether selection in a regime in which the environment changed randomly resulted in true stochastic phenotypic switching, whereas selection in other environmental switching regimes resulted in phenotypic switching rates that matched the environmental switching rate.

## 9. Can phenotypic diversity be bet hedging?

There are clearly many ways to generate phenotypic diversity, but here we focus on bet hedging because noise is often interpreted as bet hedging phenomena, especially in the microbial literature. The observation of variance among offspring in a way that may be related to their fitness may be a bet hedging strategy. The concept of bet hedging is a theoretically clear: it is a strategy (most often considered as a reproductive strategy, but conceptually bet hedging can apply to other organismal traits) that trades-off arithmetic mean fitness to maximize a genotype's multi-generational geometric mean fitness [50,51] (box 1). A genotype with the highest geometric mean fitness will win evolutionarily; geometric mean fitness is what is selected during evolution. A bet hedging strategy is one that minimizes variation in fitness across generations in the face of environmental variation [52]; maximal geometric mean fitness is achieved by an invariance in successful offspring survival and reproduction because this minimizes the risk of extinction. It is usually envisaged that variance among offspring in some trait(s) related to fitness is used to minimize their fitness variance. Phenotypic switching phenomena, including bistable phenotypic states (above), are often referred to as bet hedging strategies, apparently because of this phenotypic diversity (and, hence, probable diversity in survival and reproductive success in different environments). This, of itself, is not proof of bet hedging, because the fitness benefits of this strategy across the full range of likely environmental conditions also needs to be examined [49,53]. In these examples, if the phenotypic switching rate matches the rate at which the environments (in which each phenotypic state has maximal fitness) are encountered, then this may be maximizing both arithmetic and geometric mean fitness.

Thus, while many observations are consistent with bet hedging, they are not necessarily actual demonstrations of it; clear evidence that such observed variance among phenotypic states is an evolved and adaptive bet hedging strategy is wanting [49,53]. Demonstrations that the observed strategy has a higher geometric mean fitness compared with other strategies, when tested across relevant environments and environmental switching rates, are required. Crucially, all of the relationships between phenotypic states, relevant environments and fitness need to be understood to make such inferences. True bet hedging strategies are also difficult to observe because, by definition, their fitness consequences occur over multiple generations [52], but obviously microbial systems are very powerful in this respect. Further, other possible explanations for switching of phenotypic state also need to be considered. Key among these is phenotypic plasticity in which the environment induces the expression of the phenotypic state in question. To exclude phenotypic plasticity, the diversity and switching rate of phenotypic states that occur should, all else being equal, do so independently of the environments (external and internal) to which the genotype is exposed.

## 10. Conclusions and future directions

There have now been a significant number of studies that have observed the noise in gene expression, how gene networks can affect and be affected by noise, and some investigation of the phenotypic consequences of noise. This body of work has shown that noise can have an important role in the control of organismal phenotypes and can contribute to fitness. Overall, these studies suggest that noise in gene expression and its effects are not necessarily deleterious, but that noise can also provide a selective advantage. The major progress to date in understanding the sources of the noise in gene expression and its effects has come from studies of single-celled systems. However, noise will also occur among cells within multicellular systems, with consequent phenotypic and fitness consequences. There is therefore a need to expand the study of cellular noise to these other systems too.

A key remaining challenge is to study how evolution shapes noise. To date, most studies have manipulated gene expression noise and observed the phenotypic consequences, but fitness consequences (which may be environment specific) need to be examined too. Another approach is to seek direct evidence for selection, for example, detecting selection in the genome, and to investigate the effect of these genomic regions on gene expression noise. This would determine where in the genome selection acts with respect to noise in gene expression. This approach has the advantage of investigating evolution in natural environments, though it still requires connecting the observed noise in gene expression to fitness among environments. A powerful, complementary approach is experimental evolution in which genotypes could be selected through different environmental switching regimes, with the effect of the selection measured by direct fitness measurements, together with study of how gene expression noise has changed. A secondary way of approaching this is to experimentally select on noise in gene expression itself, using reporter gene technology [4] (box 1). More and less noisy genotypes could be evolved and the fitness consequences then measured and compared across environments.

In biological experimentation, there is always variation, which is usually addressed by having replication. Some of this variation is, of course, experimental error and not biologically interesting; however, some may well be the phenotypic consequences of noise. Natural selection selects phenotypes, and because noise can contribute to phenotypic variation of a genotype, the mechanisms underpinning noise can be moulded by evolution. Understanding what generates and maintains phenotypic variation is a key aim of evolutionary biology, and it is now clear that noise contributes significantly to phenotypic variation; if noise has an important role in evolution, we cannot afford to ignore it.
